# Mixture design-based optimization of bioactivities in steamed grain and legume blends

**DOI:** 10.1016/j.fochx.2026.103898

**Published:** 2026-04-22

**Authors:** Narae Han, Moon Seok Kang, Hana Lee, Jin Young Lee, Yu-Young Lee, Junsoo Lee, Hyun-Joo Kim

**Affiliations:** a Quality Management and Evaluation Research Division, Department of Food Sciences, National Institute of Crop Science, Rural Development Administration, Suwon 16613, the Republic of Korea; b Department of Food Science and Biotechnology, Chungbuk National University, Sheongju 28644, the Republic of Korea

**Keywords:** Antioxidants, Enzyme inhibition, Lipid metabolism, Primary metabolites, Secondary metabolites, Synergistic effect

## Abstract

A mixture design approach optimized the bioactivity of steamed blends of colored rice, sorghum, Italian millet, and black soybean. Steaming was standardized to 20 min based on preliminary lipase inhibition screening to minimize material variability. Two optimized blends showed high desirability (0.761–0.806), and predicted extraction yield and enzyme inhibition were validated within 95% prediction intervals. Blending enhanced complementary chemical attributes: black soybean improved extractability and lipid-related indices, while sorghum and colored rice provided antioxidant-rich phytochemicals. Accordingly, the optimized blends exhibited moderated antioxidant activity but markedly enhanced lipase inhibition compared with individual components, and effectively suppressed lipid accumulation in free fatty acid-treated HepG2 cells. Multivariate analysis identified flavonoids as the main drivers of antioxidant and enzyme inhibitory activities, whereas protein, free sugars, and dietary fiber were associated with lipid accumulation. These findings demonstrate that optimized blending promotes synergistic bioactivity, highlighting its potential as a multifunctional food ingredient for lipid metabolism.

## Highlights

• Mixture design identified two optimal grain-legume blends.
• Balanced lipid and amino acid composition improved nutritional quality.
• Blending enhanced extraction yield and lipase inhibition in vivo.
• Blended formulations reduced lipid accumulation in HepG2 cells.
• Flavonoid enrichment contributed to synergistic lipid-regulating effects.

## 1 Introduction

Metabolic disorders such as obesity, type 2 diabetes, and nonalcoholic fatty liver disease (NAFLD) have become major global health concerns, reflecting rapid lifestyle transitions and excessive energy intake in modern societies. Among these, NAFLD has emerged as the most common chronic liver disease, affecting approximately one–quarter of the global adult population and showing a continuous upward trend across all age groups (Riazi et al., 2022). This disease is characterized by excessive lipid accumulation in hepatocytes, independent of alcohol consumption, and is strongly associated with insulin resistance, dyslipidemia, and systemic inflammation (Younossi et al., 2023). Despite extensive clinical research, no approved pharmacological therapies are currently available, underscoring the critical need for preventive strategies based on lifestyle modifications. Dietary intervention has been recognized as a practical and sustainable approach to reducing hepatic fat deposition and improving metabolic homeostasis (Cogorno et al., 2023). Diets rich in plant-based foods, including whole grains and legumes, have been consistently linked to improved lipid metabolism and a lower incidence of metabolic syndrome, highlighting their potential role as natural modulators in the prevention of NAFLD and related disorders (Bakhshimoghaddam et al., 2024; Han, Kang, et al., 2025).

Whole grains and legumes are nutritionally balanced and rich in components that contribute to metabolic regulation. Colored rice, sorghum, and Italian millet are notable for their abundant phenolic compounds, dietary fibers, and essential amino acids (EAAs), which together promote antioxidant and lipid regulatory activities (Han et al., 2024). Colored rice varieties supply anthocyanins and γ-oryzanol that enhance oxidative stability and provide hepatic protection (Lakshmi et al., 2025). Sorghum is characterized by a high content of phenolic acids and flavonoids (Gao et al., 2025), whereas Italian millet offers balanced macronutrients, a low glycemic index, and beneficial unsaturated fatty acids (Geetha et al., 2020). Legumes, particularly black soybeans, complement these grains by providing high-quality plant proteins and isoflavones with well-documented antioxidant and lipid modulating functions (Mitharwal et al., 2024). In addition, black soybean is characterized by a lipid profile rich in unsaturated fatty acids, particularly linoleic and oleic acid, which are associated with improved lipid metabolism and cardiovascular health (Hashemirad et al., 2024). The selection of these grains and legumes was based on their complementary compositional and functional properties. While cereal grains such as colored rice, sorghum, and Italian millet are rice in phenolic compounds and dietary fiber contributing to antioxidant and enzyme inhibitory activities, black soybean provides high-quality protein, unsaturated lipids, and isoflavones that are closely associated with lipid metabolism. Accordingly, mixed-grain formulations incorporating grains and legumes are increasingly being utilized in the development of functional foods such as beverages, porridges, and ready-to-eat snack mixes, offering improve compositional diversity and functional potential compared to single-grain products (Huang & Xu, 2025). However, despite their increasing commercial and nutritional relevance, most previous studies on multigrain formulation have primarily focused on compositional profiling or individual component functionality, providing limited insight into how blending ratios influence integrated bioactivity. Therefore, studies aimed at defining optimal compositional ratios for specific functional outcomes remain limited (Li et al., 2021).

However, in practical applications, whole grains and legumes cannot be consumed or processed directly in their raw form due to their hard texture, poor digestibility, and the presence of anti-nutritional factors. Therefore, pretreatments such as boiling, roasting, popping, and steaming are essential for enhancing the edibility and bioavailability of grain-based blends (Nanje Gowda et al., 2025). Among the various techniques, steaming is recognized as a simple and effective moist-heat method that improves both the nutritional and functional qualities of grains. Steaming promotes starch gelatinization, protein digestibility, and the release of bound phenolic compounds while minimizing nutrient losses compared to dry-heat methods such as roasting (Schutte et al., 2024). Previous studies have demonstrated that steaming can increase phenolic compounds and free amino acids, thereby enhancing antioxidant capacity and lipid metabolism-related functionality (Han, Lee, et al., 2025; Narra et al., 2024). These effects are partly attributed to physicochemical and enzymatic changes induced during thermal processing. For instance, thermal treatment can activate or release endogenous cell wall-degrading enzymes (e.g., β-glucosidase and cellulase), which break down bound phenolic conjugates and polysaccharide matrices, thereby increasing the extractability of phenolic acids and flavonoids (Li et al., 2022). Concurrently, heat and moisture may cause partial denaturation of storage proteins and structural modifications of starch granules, leading to increased free amino acid content and altered susceptibility to enzyme-mediated reactions, ultimately influencing lipid metabolism-related activities (Nocente & Gazza, 2025). These processing-induced changes are expected to affect compositional interactions within multicomponent systems, particularly in relation to antioxidant and metabolic functions.

Although thermal processing is known to modify the chemical composition and bioactivity of individual grains and legumes, its role in restructuring compositional interactions within multicomponent grain-legume matrices has not been systematically addressed. Previous studies on multigrain blends have primarily reported nutritional or phytochemical profiles, with limited insight into how processing-induced matrix modifications translate into functional outcomes across different blending ratios. In particular, the combination application of steaming pretreatment and mixture design has not been explored as an analytical framework to investigate compositional interaction between primary nutrients and phytochemicals. In this study, synergy was interpreted as a potential enhancement in bioactivity arising from compositional complementarity among different components, rather than a strictly quantified deviation from additive effects. Accordingly, synergistic effects were evaluated based on integrated changes in phenolic composition, antioxidant capacity, enzyme inhibitory activities, and lipid accumulation, and should be considered as indicative rather than definitive.

Thus, the present study aimed to investigate how steaming-induced matrix modifications influence compositional interactions and bioactivity in multigrain-legume systems. To achieve this, a standardized steaming pretreatment was combined with a four-component mixture design, integrating compositional profiling, biological assays, and multivariate analysis. This approach provides a processing-informed framework for understanding composition-bioactivity relationships in multicomponent food systems.

## 2 Materials and methods

### 2.1 Plant materials and sample preparation

Colored rice (*Oryza sativa* L., cv. Boseokheukchal; BSHC) was cultivated at the Central-Northern Region Crop Research Center, Department of Crop Sciences, National Institute of Crop Science, Rural Development Administration (Suwon, South Korea). Sorghum (*Sorghum bicolor* (L.) Moench, cv. Goeunchal; GEC) and Italian millet (*Setaria italica* L., cv. Samdachal; SDC) were obtained from the Korea Agro-Fisheries & Food Trade Corporation (Seoul, South Korea). Black soybean (*Glycine max* L., cv. Soman; SM) was purchased from Anti-Cancer Food Co., Ltd. (Cheongju, South Korea). All raw materials were harvested in 2024 at their optimal maturity stage, cleaned to remove foreign matter, and stored at 10 °C under controlled conditions until use. The initial moisture contents of sorghum, colored rice, Italian millet, and black soybean were 8.58%, 10.95%, 9.00%, and 5.44%, respectively.

For the steaming treatment, each grain and legume (200 g) was rinsed three times with tap water (approximately 1:5, w/v) at 25 °C with gently manual agitation and then drained at 25 °C for 30 min to remove excess surface moisture. The SM was coarsely crushed using a blender to achieve a particle size of 3–5 mm prior to steaming, whereas the other samples were treated in wholegrain form. Steaming was carried out at 100 °C for 20 min using an electric steamer (steam wash machine, Dong-A, Hanam, South Korea) under saturated steam conditions. The samples were spread in a thin layer to ensure uniform heat transfer and consistent thermal exposure among samples. The steamed samples were immediately freeze-dried, finely ground using a grinder, and stored at −20 °C. The samples were analyzed within a short period to minimize potential oxidation and compositional changes. All samples were handled under consistent conditions to prevent degradation prior to analysis.

Analytical-grade solvents were obtained from J. T. Baker, Inc. (Phillipsburg, NJ, USA), and ultrapure water was obtained using a Milli-Q Advantage A10 purification system (Merck Millipore, Billerica, MA, USA). All other reagents and standards were purchased from Sigma-Aldrich (St. Louis, MO, USA).

### 2.2 Mixture design, optimization, and validation

A four-component mixture design was established using Design-Expert software (version 13; Stat-Ease, Minneapolis, MN, USA) to identify the optimal blending ratio of steamed grains and legume (Cornell, 2002). The experimental design focused on achieving a composition that simultaneously maximized lipase and α-glucosidase inhibitory activities as well as extraction yield. The mixture components consisted of BSHC, GEC, SDC, and SM, each varying between 0 and 100%. A {4,2} simplex lattice design was selected to effectively model the compositional interactions among the four components while maintaining experimental efficiency. This design allows for the estimation of quadratic effects, which are essential for capturing potential interactions between components, while requiring a manageable number of experimental runs compared to full factorial approaches. Accordingly, mixture combinations were generated based on a quadratic mixture model, augmented with replicates and a centroid point, resulting in total 21 experimental runs. All runs were performed in a randomized order and distributed over two independent blocks (day 1 and 2) to minimize experimental bias (Table 1). The block effect corresponding to experimental day was evaluated during model fitting; however, it was not included in the final predictive models as its contribution was negligible compared to the mixture effects (Supplementary Table S1). In this design, each vertex of the tetrahedral space represented a single component, the edge midpoints corresponded to binary mixtures, and the interior points denoted ternary or quaternary blends. Model adequacy and the significance of individual terms were evaluated using analysis of variance (ANOVA). Numerical optimization was conducted using the desirability functional approach, in which each response was converted to an individual desirability value ranging from 0 (least desirable) to 1 (most desirable). The overall desirability index was computed as the geometric mean of the individual desirability values to determine the most favorable formulation. The optimized mixtures were subsequently prepared and analyzed to validate the predicted responses. All experiments were performed with three independent formulation replicates. Each formulation included three biological replicates, and each biological replicate was analyzed in triplicate. Analytical replicates were averaged to obtain biological replicate values. And biological replicates were averaged to obtain the final formulation value.

**Table 1:** Mixture design combinations and experimental responses of blended grains treated with steaming.

Run	Block	Component 1: BSHC^1^ (%)	Component 2: GEC (%)	Component 3: SDC (%)	Component 4: SM (%)	Response 1: Yield (%)	Response 2: Lipase (%)	Response 3: α-Glucosidase (%)
1	Day 1	0	0	0	100	12.00^2^	24.65	5.51
2	Day 1	100	0	0	0	2.32	33.95	16.58
3	Day 1	0	0	50	50	6.95	24.96	14.70
4	Day 1	0	0	100	0	2.17	16.47	12.68
5	Day 1	0	0	100	0	2.11	16.14	13.06
6	Day 1	50	0	0	50	7.04	33.30	6.15
7	Day 1	25	25	25	25	4.44	30.38	12.84
8	Day 1	12.5	62.5	12.5	12.5	2.99	42.47	21.92
9	Day 1	0	0	0	100	11.77	22.11	8.88
10	Day 1	0	0	50	50	6.84	28.70	16.33
11	Day 1	50	0	50	0	2.16	25.79	16.20
12	Day 2	0	100	0	0	1.57	35.01	25.37
13	Day 2	50	50	0	0	1.88	35.10	22.66
14	Day 2	0	50	0	50	6.49	32.72	10.49
15	Day 2	62.5	12.5	12.5	12.5	3.36	33.86	10.64
16	Day 2	50	50	0	0	1.91	38.33	19.32
17	Day 2	0	100	0	0	1.54	38.62	33.34
18	Day 2	12.5	12.5	62.5	12.5	3.32	25.23	14.47
19	Day 2	100	0	0	0	2.27	32.93	22.72
20	Day 2	12.5	12.5	12.5	62.5	7.86	30.14	13.73
21	Day 2	0	50	50	0	1.94	29.67	20.29

1 BSHC, Boseokheukchal; GEC, Goeunchal; SDC, Samdachal; SM, Soman.

2 All response values represented mean of replicates (*n* = 3).

### 2.3 Determination of primary metabolites

#### 2.3.1 Protein content and amino acid composition

The crude protein content of each sample was determined using the AOAC (2019) method. Moisture content was measured by oven-drying at 105 °C under atmospheric pressure, and the nitrogen content was analyzed by the micro-Kjeldahl method using a protein analyzer (Kjeltec 2400 AUT, Foss Tector, Mulgrave, VIC, Australia). The crude protein content was calculated from the nitrogen content using specific conversion factors for each sample type: 5.95 for BSHC, 6.25 for GEC and SDC, and 5.71 for SM. All analyses were performed using standard methods with appropriate calibration using certified reference standards, and quality control was ensured through replicate measurements.

Amino acid composition was analyzed following the acid hydrolysis method described by Han, Kang, et al. (2025). Briefly, 300 mg of each powdered sample was hydrolyzed with 5 mL of 6 N HCl at 110 °C for 22 h in sealed vacuum tubes. After hydrolysis, the reaction mixtures were passed through filter paper, and the filtrates were quantitatively adjusted to 40 mL with ultrapure water in volumetric flasks. The resulting hydrolysates were further diluted 20-fold with water before injection into an automatic amino acid analyzer (LA8900, Hitachi High-Tech Co., Tokyo, Japan). The amino acids were identified and quantified using an amino acid mixture standard solution (Type H; Wako Pure Chemicals, Osaka, Japan). The constituent amino acid content was expressed in grams per 100 g of protein. Tryptophan was not determined due to its degradation under acid hydrolysis conditions. Methionine and cysteine were quantified; however, minor analytical limitations associated with acid hydrolysis should be considered. The EAA content was calculated as the sum of histidine, lysine, phenylalanine, threonine, methionine, isoleucine, leucine, and valine. The non-EAAs (NEAAs) included alanine, arginine, aspartic acid, glutamic acid, cysteine, glycine, proline, serine, and tyrosine. The branched-chain amino acids (BCAAs) were represented by total isoleucine, leucine, and valine, while the sulfur-containing amino acids (SAAs) were calculated as the sum of methionine and cysteine.

#### 2.3.2 Carbohydrate content and composition

The total carbohydrate content was calculated by difference according to the AOAC (2019) as follows: Carbohydrate (g per 100 g dry weight) = 100 − (moisture + ash + crude protein + crude lipid). As this approach may propagate analytical errors, the values should be interpreted with caution.

The total starch content was determined using a Total Starch Assay Kit (K-TSTA, Megazyme, Bray, Ireland) based on enzymatic hydrolysis with thermostable α-amylase and amyloglucosidase, following the manufacturer's instructions. The absorbance of released glucose was measured at 510 nm.

Dietary fiber content was determined by the enzymatic-gravimetric method using a Fibertec System (SE/Fibertec 1023, Foss, Hillerød, Denmark), as described in the AOAC (2019). The total dietary fiber content was calculated as the sum of the insoluble and soluble dietary fiber contents.

For the free sugar analysis, 1 g of powdered sample was extracted with 10 mL of 70% ethanol at 24 °C for 15 h with occasional shaking. The extract was centrifuged, and the supernatant was filtered through a 0.2 μm syringe filter prior to high-performance liquid chromatography (HPLC) analysis (Chromaster, Hitachi Ltd., Tokyo, Japan). Free sugars were separated on an NH_2_ column (250 × 4.6 mm, 5 μm, Hitachi Ltd.) using 70% acetonitrile as the mobile phase. Detection was performed using a refractive index detector. Glucose, fructose, sucrose, raffinose, and stachyose were identified and quantified based on retention times and calibration curves of the corresponding commercial standards.

#### 2.3.3 Lipid content and fatty acid composition

The crude lipid content was determined according to the AOAC method using a Soxhlet extractor (Soxtec System HT 1043 extraction unit; Foiss Tecator, Hoganas, Sweden) with diethyl ether as the extraction solvent (AOAC, 2019).

For fatty acid analysis, 2 g of each powdered sample was mixed with 20 mL of chloroform-methanol (2:1, v/v) containing 0.01% butylated hydroxytoluene (BHT) and shaken for 1 h in an ice bath. After centrifugation, the residue was re-extracted with 10 mL of the same solvent, and both extracts were combined. The combined organic phase was washed with 7.5 mL of 0.88% NaCl solution and kept at 4 °C for 30 min to allow phase separation. The lower chloroform layer was carefully collected and passed through anhydrous Na_2_SO_4_ for dehydration. The filtrate was evaporated under nitrogen, and the lipid residue was weighed to calculate the total lipid content. Fatty acid composition was determined after conversion to fatty acid methyl esters (FAMEs). The lipid solution (50 mg lipid equivalent) was treated sequentially with 1.5 mL of 0.5 M methanolic NaOH (85 °C, 10 min) and 2 mL of 14% BF_3_-MeOH (85 °C, 3 min). After cooling, 2 mL of isooctane containing 0.01% BHT and 1 mL of saturated NaCl were added, and the mixture was vortexed for 1 min. The upper isooctane layer containing FAMEs was passed through an anhydrous Na_2_SO_4_ bed. The eluate was adjusted to a final volume of 3 mL with isooctane, filtered through a 0.2 μm PTFE syringe filter, and analyzed by gas chromatography (GC) (Agilent 8890 GC System, Agilent Technologies, Santa Clara, CA, USA). GC analysis was carried out following the procedure of Han, Kang, et al. (2025), and individual FAMEs were identified and quantified using commercial standard mixtures (Supelco 37 Component FAME Mix, Supelco, Bellefonte, PA, USA).

### 2.4 Determination of secondary metabolites

#### 2.4.1 Total polyphenol (TPC) and flavonoid contents (TFC)

The ethanolic extracts for the determination of TPC and TFC were prepared according to the method described by Han, Lee, et al. (2025). Briefly, 3 g of each powdered sample was extracted with 70% ethanol while shaking for 24 h at 25 °C. The extracts were filtered and concentrated under reduced pressure until completely dry, and the residues were weighed to calculate the extraction yield. The dried residues were reconstituted in dimethyl sulfoxide (DMSO) at a concentration of 100 mg/mL and used for subsequent analyses. TPC was determined using the Folin–Ciocalteu method (Slinkard & Singleton, 1977), and TFC was analyzed using the aluminum chloride colorimetric method (Yu et al., 2005), as previously described. TPC and TFC were expressed as mg gallic acid equivalents (GAE) per g dry weight (DW) and mg catechin equivalents (CE) per g DW, respectively.

#### 2.4.2 Quantitative analysis of individual phenolic compounds by HPLC

Individual phenolic compounds were analyzed using HPLC (Chromaster, Hitachi Ltd.), following the method described by Han et al. (2024) with slight modifications. The ethanolic extracts, prepared as described above, were diluted with 15% acetonitrile to a concentration of 1 mg/mL prior to injection. The analysis was performed on a C18 column (250 × 4.6 mm, 5 μm, Agilent Technologies) using a diode array detector. The mobile phase consisted of (A) water containing 0.1% formic acid and (B) acetonitrile containing 0.1% formic acid, at a flow rate of 0.8 mL/min. The gradient elution program was as follows: 0 min, 95% A; 5 min, 90% A; 10 min, 85% A; 20 min, 75% A; 30 min, 65% A; 40 min, 50% A; 45 min, 20% A; and 50 min, 95% A. Detection was monitored simultaneously at 280, 320, and 360 nm. Phenolic compounds in the extracts were identified by comparing their retention times and UV spectra with those of authentic standards and were quantified using calibration curves constructed from the corresponding standards.

### 2.5 Evaluation of biological activities

#### 2.5.1 Antioxidant activities

The antioxidant activities of the ethanolic extracts were evaluated based on their radical scavenging capacity and ferric reducing antioxidant power (FRAP). The 2,2-diphenyl-1-picrylhydrazyl (DPPH) radical scavenging activity was determined by mixing 20 μL of the diluted extract with 200 μL of 0.2 mM DPPH solution. The reaction mixture was incubated at 25 °C for 30 min, and the absorbance was read at 520 nm using a microplate reader (Elx 808, BioTek Instruments Inc., Winooski, VT, USA). The 2,2′-azinobis(3-ethylbenzothiazoline-6-sulfonic acid) (ABTS) radical scavenging activity was analyzed by combining 20 μL of the diluted extract with 200 μL of 7.4 mM ABTS solution, followed by incubation at 25 °C for 30 min and absorbance measurement at 734 nm. The FRAP assay was conducted by mixing 6 μL of diluted extract with 180 μL of FRAP reagent and 18 μL of ultrapure water. After incubation at 25 °C for 10 min, the absorbance was measured at 593 nm. DPPH and ABTS scavenging activities are expressed as mg Trolox equivalents (TE) per g DW and FRAP was expressed as mM per g DW.

#### 2.5.2 Enzyme inhibitory activities

To evaluate the potential effects of the extracts on lipid metabolism associated with NAFLD, the inhibitory activities of pancreatic lipase and α-glucosidase were determined (Zhang et al., 2024). Lipase inhibitory activity was determined using the enzymatic method described by Hashemirad et al. (2024) with minor modifications. The ethanolic extract prepared above was diluted with assay buffer (50 mM Tris-HCl containing 0.1 mM CaCl_2_, pH 7.2) to a concentration of 5 mg/mL. Subsequently, 20 μL of the diluted extract was mixed with 160 μL of porcine pancreatic lipase Type II solution (0.2 U/mL in assay buffer) and incubated at 37 °C for 15 min. The reaction was initiated by adding 20 μL of 1 mM *p*-nitrophenyl butyrate as the substrate and allowed to proceed for 30 min at 37 °C. The absorbance was recorded at 410 nm using a microplate reader. Orlistat was used as a positive control (PC) (half-maximal inhibitory concentration [IC_50_] = 0.56 μg/mL).

For the determination of α-glucosidase inhibitory activity, the ethanolic extracts prepared above were diluted to 10 mg/mL with the assay buffer (0.1 M sodium phosphate buffer, pH 7.0). Then, 10 μL of the extract was mixed with 90 μL of α-glucosidase solution (0.5 U/mL in assay buffer) and pre-incubated at 37 °C for 20 min. Subsequently, 100 μL of 1.5 mM *p*-nitrophenyl-α-D-glucopyranoside was added as a substrate, and the mixture was further incubated for 6 min at 37 °C in the dark. Absorbance was recorded at 405 nm using a microplate reader. Acarbose was used as a PC (IC_50_ = 26.47 mM/mL).

### 2.6 Inhibitory effects on lipid accumulation in HepG2 cells

The inhibitory effect of the extracts on lipid accumulation was evaluated using the Oil Red O staining assay, following the procedure described by Han, Lee, et al. (2025). HepG2 cells (human hepatocellular carcinoma, ATCC, Manassas, VA, USA) were maintained in Dulbecco's modified Eagle's medium supplemented with 10% fetal bovine serum, 100 U/mL penicillin, and 50 μg/mL streptomycin at 37 °C in a humidified 5% CO_2_ incubator. Cytotoxicity was assessed using the MTT assay. Briefly, cells were seeded in 96-well plates and incubated for 24 h, followed by treatment with the sample extracts and a free fatty acid (FFA) mixture (oleate:palmitate = 2:1). After 24 h, 0.15 mg/mL MTT solution was added to each well and incubated for 2 h. The resulting formazan crystals were dissolved in DMSO, and the absorbance was read at 550 nm using a microplate reader. No cytotoxicity was observed at a concentration of 100 μg/mL, which was therefore selected for subsequent lipid accumulation analysis. For the lipid accumulation assay, HepG2 cells were treated with the extracts (100 μg/mL) and FFA mixture (500 μM) for 24 h. After fixation with 10% formalin, the cells were stained with 0.5% Oil Red O solution for 10 min. The stained lipids were eluted with isopropanol, and the absorbance was measured at 490 nm.

### 2.7 Statistical and multivariate analyses

All data were expressed as the mean ± standard deviation (SD, *n* = 3). Statistical analyses were conducted using SigmaPlot version 14.0 (Systat Software, San Jose, CA, USA) and SPSS Statistics software (version 18.0; SPSS Inc., Chicago, IL, USA). A one-way ANOVA was performed using the general linear model procedure, and significant differences among the means were identified using Tukey's honest significance test (HSD) test at a significance level of *p* < 0.05. Integrated data clustering and visualization of the experimental results were carried out using MetaboAnalyst 6.0 (https://www.metaboanalyst.ca/; accessed October 15, 2025) (Pang et al., 2024).

## 3 Results and discussion

### 3.1 Preliminary standardization of steaming pretreatment

Prior to mixture design, the steaming treatment conditions were standardized to minimize variations arising from individual steamed materials. Each grain and legume was steamed for 0, 10, 20, 30, 40, or 50 min, and the resulting extracts were evaluated for lipase inhibitory activity (Supplementary Fig. 1). The responses varied among the materials: GEC showed a progressive increase in inhibition up to 20 min, followed by a gradual decline, whereas SDC exhibited a continuous increase for up to 50 min. BSHC and SM showed the highest inhibition at 20 and 10 min, respectively, with no significant changes at longer steaming durations. Overall, steaming for 20 min enhanced lipase inhibitory activity compared to that of the untreated control across all materials.

To determine the appropriate pretreatment conditions for mixture design, two approaches were compared: (i) applying a uniform steaming condition (20 min) to all samples, and (ii) applying an individual condition, in which each material was steamed for its optimal duration showing the highest activity (BSHC, 30 min; GEC, 20 min; SDC, 50 min; and SM, 30 min). Preliminary mixture design modeling indicated that the predicted lipase inhibitory activity was lower under the individual condition (29.10–33.71%) than under the uniform 20-min condition (33.69–35.72%). Considering both statistical consistency and practical feasibility, the uniform 20 min steaming condition was selected for subsequent experiments. This standardized pretreatment enabled a fair comparison of the compositional and biological responses among the blends and ensured reliable model fitting in the subsequent mixture design. This standardization step served as a preliminary screening based on lipase inhibition, whereas the subsequent mixture optimization incorporated multiple response variables, including extraction yield and α-glucosidase inhibition.

### 3.2 Mixture design and model validation

The optimal blending ratios of BSHC, GEC, SDC, and SM were determined using a mixture design approach. A mixture design approach based on RSM was employed to systematically model and optimize the blending ratios of steamed grains–legume mixtures. RSM provides a statistically rigorous framework to quantify the main and interaction effects among compositional variables, allowing for the development of predictive polynomial models that relate formulations to functional outcomes (Li et al., 2021). This approach has been recognized as an effective tool for elucidating compositional synergies in multi-component food systems, providing both scientific insights and practical guidance for functional product design (Han, Kang, et al., 2025). In the present study, the RSM-based mixture design enabled the evaluation of composition–dependent functional responses in steamed grain–legume blends, thereby identifying the optimal ratio that maximizes integrated bioactivities.

Table 1 presents the experimental responses for extraction yield and lipase and α-glucosidase inhibitory activities, which ranged from 1.54–12.00%, 16.14–42.47%, and 5.51–33.34%, respectively. ANOVA results (Supplementary Table 1) indicated that linear, quadratic, and cubic models were suitable for predicting yield, lipase, and α-glucosidase inhibition, respectively. Model fit was supported by high F–vales (yield: 6252.21; lipase: 32.50; α-glucosidase: 6.17) and a significant *p*-value (*p* < 0.05). All models exhibited high R^2^ and adjusted R^2^ values, with adequate precision exceeding four, confirming their predictive performance. The regression equations of the model in terms of the coded factors are as follows:

Yield=2.28A+11.74B+1.57C+2.11D

$$1/\left(\text{Lipase}\right)=0.0299A+0.0432B+0.0265C+0.0615D-0.0219\mathrm{AB}-0.0049\;\mathrm{AC}-0.0246\mathrm{AD}-0.0185\mathrm{BC}-0.0574\mathrm{BD}-0.0435\mathrm{CD}$$

α−glucosidase=19.62A+9.18B+27.31C+14.85D−25.45AB−18.21AC+3.4AD−39.58BC+21.8BD−11.73CD+430.36ABC−905.6ABD−291.72ACD+795.83BCD

The optimization criteria targeted the maximization of lipase inhibitory activity, along with concurrent improvements in α-glucosidase inhibition and extraction yield. The optimization criteria targeted the maximization of lipase inhibitory activity, along with concurrent improvements in α-glucosidase inhibition and extraction yield. Six candidate solutions were generated, among which candidate formulations 1 (B1) and 3 (B2) were selected as the representative optimal blends. B1 consisted of 38.835% GEC, 15.978% SDC, and 45.187% SM, exhibiting the highest desirability score (0.806), whereas B2 comprised 30.712% BSHC, 33.563% GEC, and 35.725% SM, showing a slightly lower desirability (0.761) (Supplementary Table 2). Although B2 had a marginally lower desirability value than candidate formulation 2, it was selected to include BSHC, thereby representing broader compositional diversity among the grain and legume blends. This selection enabled a comparative evaluation between the most statistically optimized blend and another composition incorporating a different grain type, thus enhancing the interpretability and practical relevance of the optimization results. Desirability values ranging from 0 to 1 reflect the closeness of the predicted responses to the target goals; a score of 1 denotes perfect fulfillment of all criteria, while 0 indicates a completely undesirable outcome (Akinoso et al., 2024). In recent optimization studies, desirability values between 0.37 and 1.00 have been considered acceptable (Patel & Wairkar, 2025). In this context, the desirability scores obtained in our study fell within the acceptable to excellent range, confirming that the developed models provided reliable and practically relevant optimization outcomes. The predicted responses for B1 and B2 were 6.25% and 5.42% yield, 34.06% and 35.50% lipase inhibitory activity, and 33.34% and 24.90% α-glucosidase inhibitory activity, respectively (Table 2). Experimental validation showed that observed values fell within the 95% prediction interval, supporting the reliability of the developed model. These results demonstrated that the mixture design approach effectively optimized the bioactive potential of steamed grain and legume blends by leveraging the complementary properties of the individual components.

**Table 2:** Experimental validation of the optimized grain mixtures treated with steaming.

(A) Optimized blend 1 (B1)					
Response	Predicted value		Observed value		
	Mean	Std Dev^1^	95% PI^2^ low	Data Mean	95% PI high
Yield (%)	6.25	0.10	6.11	6.34	6.39
Lipase (%)	34.06	2.69	29.23	33.95	40.21
α-Glucosidase (%)	33.34	2.85	9.55	32.48	57.13

**(B) Optimized blend 2 (B2)**					
**Response**	**Predicted value**		**Observed value**		
	**Mean**	**Std Dev**	**95% PI low**	**Data Mean**	**95% PI high**
Yield (%)	5.42	0.10	5.29	5.48	5.55
Lipase (%)	35.50	2.92	30.53	35.29	41.72
α-Glucosidase (%)	24.90	2.85	−8.17	25.87	57.97

1 Std Dev, standard deviation.

2 The 95% Prediction Interval (95% PI) represents the range in which the predicted values for a given composition is expected to fall with a 95% probability in actual experiments.

### 3.3 Primary metabolites

Primary metabolites, such as proteins, carbohydrates, and lipids, play pivotal roles in hepatic lipid metabolism and systemic energy homeostasis. Imbalances in these macronutrients are closely associated with the onset and progression of NAFLD. In particular, amino acid parameters such as the EAA/NEAA ratio, BCAAs, and SAA are known to modulate hepatic lipogenesis and β-oxidation, thereby mitigating lipid accumulation in hepatocytes (Liao et al., 2024; Surugihalli et al., 2023; Vanweert et al., 2022). Carbohydrate and lipid-related indices, including starch and dietary fiber content, the polyunsaturated fatty acid/saturated fatty acid (PUFA/SFA) and Omega-6/Omega-3 ratios, also affect hepatic glucose flux, lipid oxidation, and inflammatory balance (Al-Taher & Nemzer, 2023; Hashemirad et al., 2024; Ni et al., 2023). Therefore, compositional profiling of these primary metabolites was conducted to clarify how the nutritional composition of steamed grain–legume blends contributes to their potential role in modulating hepatic lipid accumulation.

**Fig. 1:**
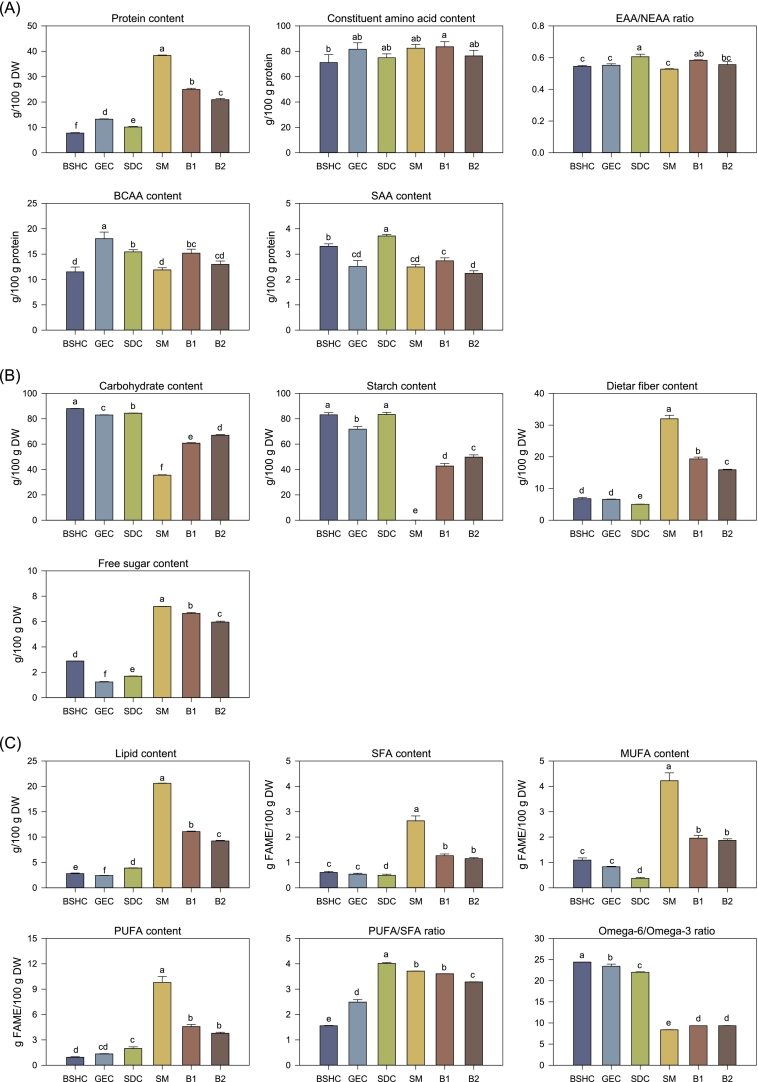
Changes in primary metabolites of individual components and optimized blends. BSHC, GEC, SDC, SM, B1, and B2 refer to Boseokheukchal, Goeunchal, Samdachal, Soman, optimized blend 1, and optimized blend 2, respectively. Different letters above bars indicate significant differences at *p* < 0.05 (Tukey's honest significance [HSD] test). (A) Protein-related indices including crude protein content, constituent amino acid content, essential amino acid/non-essential amino acid (EAA/NEAA) ratio, branched-chain amino acid (BCAA) content, and sulfur-containing amino acid content. (B) Carbohydrate-related indices, including the contents of crude carbohydrates, starch, dietary fiber, and free sugars. (C) Lipid-related indices, including the contents of crude lipids, saturated fatty acids (SFA), monounsaturated fatty acids (MUFA), and polyunsaturated fatty acids (PUFA), as well as the ratios of PUFA/SFA and omega-6/omega-3 fatty acids.

Crude protein content (Fig. 1A) varied considerably among the individual components. SM had the highest content (38.33 g/100 g DW), followed by GEC (13.21 g/100 g DW). B1 and B2 had lower protein contents than SM alone but higher levels than the other individual components, indicating that blending with SM improved the overall protein composition of the formulations. The constituent amino acid content of all samples ranged from approximately 71.15 to 83.54 g/100 g protein. When comparing the amino acid composition by functional group, the EAA/NEAA ratio was highest in SDC (0.61), whereas the other components showed lower values ranging from 0.53 to 0.55. The total BCAA content was significantly higher in the millet-type grains, GEC (18.05 g/100 g DW) and SDC (15.43 g/100 g DW), than in BSHC and SM (*p* < 0.05). Similarly, the total SAA content was greatest in SDC (3.72 g/100 g DW), followed by BSHC (3.31 g/100 g DW). Despite having the highest protein content, SM exhibited the lowest EAA/NEAA ratio, BCAA content, and SAA content among all individual components. The blended samples showed intermediate values compared to those of the individual components. Notably, B1 presented a relatively higher EAA/NEAA ratio (0.58), BCAA content (15.21 g/100 g DW), and SAA content (2.74 g/100 g DW) than B2, which may be attributed to the inclusion of SDC in its formulation. Although SM exhibited the highest protein content, blending with other grains slightly reduced the absolute protein level but improved the overall quality. The incorporation of GEC and SDC, which are rich in functionally valuable amino acids, complemented the amino acid profile of SM, resulting in a more balanced composition in B1 and B2 with a relatively higher EAA/NEAA ratio, and BCAA and SAA contents. Such compositional improvements are nutritionally meaningful because amino acid quality, rather than total protein content, has been associated with enhanced hepatic lipid metabolism.

Distinct differences in carbohydrate composition were observed between SM and other grains (Fig. 1B). The total carbohydrate content of SM was 35.55 g/100 g DW, markedly lower than that of the other components (82.98–88.00 g/100 g DW). In BSHC, GEC, and SDC, starch accounted for approximately 90% of total carbohydrates, whereas in SM, nearly 90% of the carbohydrates consisted of dietary fiber. This pattern is consistent with established compositional differences between grains and legumes; in grains, carbohydrates typically represent approximately 60–70% of seed DW and are predominantly starch, reflecting endosperm starch biosynthesis (Lafiandra et al., 2014). In contrast, legumes have lower starch content and a higher proportion of non-starch polysaccharides and oligosaccharides, yielding substantially greater dietary fiber (Kumar et al., 2025). Accordingly, the markedly starch-dominant profile of BSHC, GEC, and SDC and the fiber-dominant profile of SM conform to these general trends. Similarly, differences in carbohydrate composition were reflected in the free sugar content of the individual components (Supplementary Table 3). SM contained the highest amount (7.19 g/100 g DW), followed by BSHC, SDC, and GEC. The composition pattern of free sugars also differed substantially between SM and the other grains. In BSHC, GEC, and SDC, the majority of free sugars were mono- and disaccharides, with only minor proportions of oligosaccharides (0.02, 0.10, and 0.15 g/100 g DW, respectively). In contrast, SM exhibited a markedly higher level of oligosaccharides (6.09 g/100 g DW), compared with 1.10 g/100 g DW for mono- and disaccharides, reflecting its typical legume-type carbohydrate profile dominated by raffinose-family oligosaccharides. This composition indicates a predominance of slowly digestible carbohydrates in SM, primarily raffinose-family oligosaccharides, which may contribute to attenuated postprandial glycemic responses, reduced hepatic lipid accumulation, and favorable modulation of the gut microbiota (Liu et al., 2024; Ni et al., 2023). Accordingly, SM has a carbohydrate profile that is potentially advantageous for the dietary prevention of NAFLD. For the blended samples B1 and B2, which contained 45.187% and 35.725% SM, respectively, the overall carbohydrate profiles were intermediate between those of SM and the other grains, indicating that blending proportionally balanced starch- and fiber-rich components across the formulations. Although the total starch content of the blended samples was slightly higher than that of SM alone, their dietary fiber and oligosaccharide levels remained higher than those of the other components. This suggests that the functional carbohydrate characteristics of SM were largely retained and transferred to the mixture through blending. From the perspective of carbohydrate composition, SM exhibited the most favorable potential for NAFLD prevention, wherea the blended samples demonstrated improved carbohydrate profile that may still contribute positively to metabolic health compared to the consumption of single cereal grains.

Total lipid content varied markedly among the samples. As a legume, SM exhibited a distinctly higher lipid content (20.60 g/100 g DW) than the other grains (all below 4%). Linoleic, oleic, and palmitic acids were the predominant fatty acids detected across all individual components, constituting the characteristic lipid composition commonly observed in grain and legume matrices (Supplementary Table 3) (Han, Kang, et al., 2025). Correspondingly, SM contained a greater proportion of SFAs, monounsaturated fatty acids (MUFAs), and PUFAs than the cereal components. B1 and B2 also showed elevated lipid content due to the inclusion of SM, exhibiting intermediate values between SM and the other grains. The PUFA/SFA ratio, an indicator of lipid quality and cardiovascular health, was highest in SDC (4.01) and SM (3.71), followed by GEC (2.49) and BSHC (1.55). The PUFA/SFA ratio, an established indicator of lipid nutritional quality and cardiovascular health, reflects the balance between unsaturated fatty acids and SFA in dietary lipids. Ratios above 0.45 are considered desirable for maintaining optimal plasma lipid profiles and reducing the risk of metabolic disorders (Hashemirad et al., 2024). However, excessively high values (> 1.0) may decrease oxidative stability and shelf-life due to the susceptibility of PUFA to lipid peroxidation (Millao et al., 2023). Based on the fatty acid composition data reported in previous studies, the PUFA/SFA ratio for colored rice, sorghum, Italian millet, and black rice can be approximately estimated as 1.48–1.62 (Zhao et al., 2025), 1.65–3.14 (Pontieri et al., 2022), 4.48–6.02 (Choi et al., 2019), and 3.67–4.63 (Li et al., 2024), respectively. These estimated ranges are comparable to those obtained in this study. This consistency suggests that the fatty acid composition of our materials aligns well with the previously characterized profiles of grains and legumes. Overall, the PUFA/SFA ratio in individual components is typically greater than 1.0, indicating a predominance of unsaturated fatty acids and generally favorable lipid quality, although a high PUFA content may reduce oxidative stability during processing and storage. Notably, blending slightly moderated the PUFA/SFA ratio relative to individual grains, suggesting that the mixture formulation could improve lipid stability while maintaining favorable nutritional quality. The Omega-6/Omega-3 ratio ranged from 8.39 (SM) to 24.40 (BSHC), which is typical for cereal and legume matrices. A range of 4–10 is often suggested in the literature as favorable for maintaining the physiological balance between pro- and anti-inflammatory eicosanoids (Simopoulos, 2002). High Omega-6/Omega-3 ratios are associated with a pro-inflammatory lipid profile and an increased risk of metabolic disorders, whereas lower ratios favor an anti-inflammatory balance (Al-Taher & Nemzer, 2023). Importantly, B1 and B2, which were influenced by the inclusion of SM, exhibited Omega-6/Omega-3 ratios below 10, indicating that blending effectively adjusted the fatty acid composition to a nutritionally favorable range. Collectively, these findings indicate that the mixture design approach not only optimizes bioactivity but also improves nutritional lipid quality through the balanced integration of grains and legumes. Considering that alterations in lipid metabolism are closely linked to the progression and mitigation of NAFLD, the observed differences in fatty acid composition among the samples are likely to be associated with their biological activities. In particular, high PUFA/SFA and balanced Omega-6/Omega-3 ratios are associated with improved membrane fluidity, enhanced antioxidant defense, and attenuation of hepatic lipid accumulation (Simopoulos, 2002). Therefore, it is plausible that blends with more favorable lipid profiles also display stronger inhibitory activities against lipid-related enzymes and cellular lipid deposition, as discussed in the following section. These findings suggest that amino acid quality and lipid indices contribute to hepatic lipid regulation through modulation of lipogenesis, β-oxidation, and membrane lipid remodeling. The carbohydrate profiles of the blend, particularly the higher dietary fiber and raffinose–family oligosaccharides, likely reduce lipid accumulation by influencing FFA uptake and hepatic glucose–lipid flux. Collectively, the primary metabolites appear to drive the basal metabolic tone that underlies the lipid-lowering capacity of the blended formulations.

### 3.4 Secondary metabolites

**Fig. 2:**
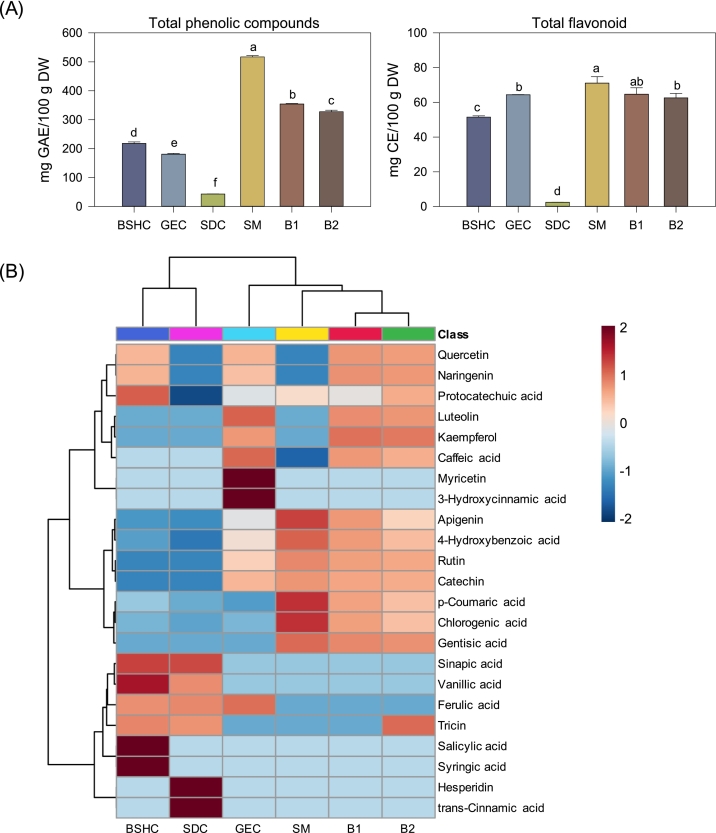
Changes in secondary metabolites (phenolic compounds) of individual components and optimized blends. BSHC, GEC, SDC, SM, B1, and B2 refer to Boseokheukchal, Goeunchal, Samdachal, Soman, optimized blend 1, and optimized blend 2, respectively. Different letters above bars indicate significant differences at *p* < 0.05 (Tukey's HSD range test). (A) Total phenolic content and total flavonoid content. (B) Heatmap visualization of individual phenolic compounds quantified by HPLC. The color intensity represents the relative abundance of each compound across samples.

The extraction yields of BSHC, GEC, SDC, and SM were 2.27%, 1.48%, 2.03%, and 11.47%, respectively, indicating that SM exhibited the highest extractability among the individual components. Consequently, the yields of the blended samples were intermediate, with B1 and B2 showing 6.37% and 5.48%, respectively (data not shown). The TPC of the individual components followed the order SM (517.06 mg GAE/100 g DW) > BSHC (217.98) > GEC (180.79) > SDC (43.09) (Fig. 2A). The high TPC of SM was largely attributed to its high extraction yield. When normalized to the extract weight, however, GEC (122.08 mg GAE/g extract) and BSHC (96.16 mg GAE/g extract) showed 2.71- and 2.13-fold higher TPC than SM, respectively. For the TFC, GEC and BSHC exhibited the highest proportions relative to TPC (35.35% and 23.63%, respectively), whereas SM had a comparatively lower ratio (13.74%). These results are consistent with previous reports identifying sorghum and colored rice as phenolic-rich grains with high flavonoid accumulation but limited extractability (Han et al., 2024). B1 and B2 presented intermediate TPC and TFC levels between those of grains and legume. This indicates that blending effectively combined the high extractability of SM with the phenolic richness of GEC and BSHC, thereby improving both extraction efficiency and functional compound yield. This compositional balance highlights the compositional advantage of the mixture design approach.

To obtain a comprehensive understanding of phenolic enrichment, both total contents (TPC and TFC) and compound-specific profiles were evaluated. A total 23 phenolic compounds were identified by HPLC and visualized as a heatmap (Fig. 2B), classified into hydroxybenzoic acids, hydroxycinnamic acids, and flavonoids. Among the individual components, BSCH and SDC formed a distinct cluster characterized by high levels of sinapic acid, vanillic acid, ferulic acid, and tricin, representing hydroxycinnamic acid-rich phenolic patterns. Within this cluster, BSHC contained markedly higher amounts of salicylic acid and syringic acid, whereas SDC showed higher levels of hesperidin and trans-cinnamic acid. SM was enriched in apigenin, 4-hydroxybenzoic acid, rutin, catechin, p-coumaric acid, chlorogenic acid, and gentisic acid, while GEC showed a wider diversity of hydroxycinnamic acids and flavonoid compounds, albeit at lower absolute concentrations. The blended samples (B1 and B2) exhibited intermediate or slightly higher total phenolic concentrations than the individual components, suggesting a compositional complementarity between legume and cereal grains.

The blending effect was particularly pronounced in flavonoid subclasses, where both B1 and B2 displayed higher levels of quercetin, naringenin, and kaempferol than any single component. Such increases were not observed for hydroxybenzoic or hydroxycinnamic acids, indicating that flavonoid-type compounds were likely major contributors to the observed increase of the phenolic content after blending. This enhancement may be attributed to matrix interactions during co-steaming (Nocente & Gazza, 2025). Heat–induced unfolding of SM proteins can exposes binding sites that interact with flavonoids, weakening their association with polysaccharide–lignin complexes and promoting the release (Kieserling et al., 2024). In addition, unsaturated lipid from SM may create a transient lipid-aqueous interface that enhances flavonoid solubilization (Han, Lee, et al., 2025; Li et al., 2022; Nocente & Gazza, 2025). These combined interactions likely facilitate both the liberation and stabilization of flavonoids. Overall, the integration of phenolic-rich grains and high-extractability legume broadened the phenolic diversity of the blends, contributing to enhanced antioxidant and enzyme inhibitory potential. These findings suggest that phenolic subclass distribution, rather than total phenolic content alone, is a key determinant of functional improvement in blended system. Such compositional complementation may also underlie the improved biological activities of the blends. Flavonoids (e.g., quercetin, kaempferol, and naringenin) and hydroxycinnamic (e.g., chlorogenic and p-coumaric acids) are well recognized for their antioxidant and lipid-modulating properties. The enrichment of flavonoids in the blends suggests enhanced interactions with lipid-hydrolyzing enzymes and reactive oxygen species (ROS)-associated pathways. These compounds are known to modulate active-site residues, inhibit lipid micelle formation, and stabilize redox states, thereby directly contributing to antioxidant and enzyme inhibitory responses (Huang et al., 2020; Mahboob et al., 2023). Thus, the phenolic subclass distribution, rather than just the total phenolic content, is a key determinant of the functional improvements observed after blending.

### 3.5 Biological activity

**Fig. 3:**
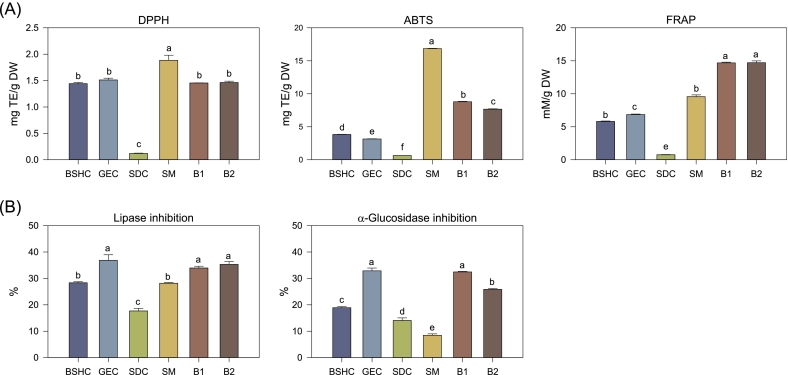
Antioxidant and enzyme inhibitory activities of individual components and optimized blends. BSHC, GEC, SDC, SM, B1, and B2 refer to Boseokheukchal, Goeunchal, Samdachal, Soman, optimized blend 1, and optimized blend 2, respectively. Different letters above bars indicate significant differences at *p* < 0.05 (Tukey's HSD test). (A) 2,2-Diphenyl-1-picrylhydrazyl (DPPH) and 2,2′-azinobis(3-ethylbenzothiazoline-6-sulfonic acid) (ABTS) radical-scavenging activities and ferric reducing antioxidant power (FRAP). (B) Lipase and α-glucosidase inhibitory activities of the extracts at the specified assay concentrations (lipase: 5 mg extract/mL; α-glucosidase: 10 mg extract/mL). Orlistat and acarbose were used as positive controls (PCs) for lipase and α-glucosidase assays, respectively.

The changes in antioxidant activity according to blending are presented in Fig. 3A. Among the individual components, SM exhibited the highest antioxidant capacity in all assayss (DPPH, ABTS, and FRAP), followed by GEC and BSHC, while SDC exhibited the lowest activity. Consistent with the TPC and TFC results, the strong antioxidant response of SM appeared to be associated with its high extraction yield rather than its inherently high phenolic concentration. When recalculated on an extract-weight basis, GEC demonstrated markedly higher specific activities (DPPH: 102.08 mg TE; ABTS: 213.13 mg TE; FRAP: 462.89 mM) compared with SM (DPPH: 16.43 mg TE; ABTS: 146.90 mg TE; FRAP: 83.39 mM), confirming that GEC possesses a high intrinsic antioxidant potential, while SM primarily contributes to extraction efficiency and overall yield. B1 and B2 exhibited radical-scavenging activities at levels intermediate between those of the individual components; however, their FRAP values were significantly enhanced relative to those of any single component. This improvement suggests a potential synergistic redox effect arising from the complementary characteristics of GEC and SM, with the former supplying phenolic-rich antioxidants and the latter enhancing the extractability and solubility of redox-active compounds (Chen et al., 2022). Such cooperative behavior between legume and cereal grain matrices highlights the potential for optimized blending to maximize antioxidant performance through both compositional and physicochemical interactions.

The inhibitory activities against pancreatic lipase and α-glucosidase are shown in Fig. 3B. Among the individual components, GEC exhibited the greatest inhibition of both enzymes (36.38% and 32.88%, respectively), followed by BSHC (28.35% and 18.90%, respectively). SM showed moderate lipase inhibition (21.18%), which was higher than that of SDC (17.69%), but its α-glucosidase inhibition (8.46%) was lower than that of SDC (14.04%). These results indicate that GEC and BSHC, which are rich in flavonoids and hydroxycinnamic acids, predominantly contribute to the enzyme inhibitory potential of the individual components. B1 and B2 exhibited lipase inhibitory activities of 33.95% and 35.31%, respectively, comparable to that of GEC, whereas α-glucosidase inhibition was slightly lower in B2 (25.87%) than in B1 (32.48%). Considering that the enzyme inhibitory activities were evaluated on an extract-weight basis rather than per gram of dry sample, the enhanced extraction yield in the blended samples suggests that their actual inhibitory potential per unit of raw material may be increased than that of any single component (Tourabi et al., 2023). This implies that blending not only maintained the high enzyme-inhibitory efficiency of GEC but also improved extractability through the inclusion of SM, thereby enhancing the overall functional efficacy per processing unit. As described above, this effect may be associated with matrix-assisted interactions between SM-derived macromolecules (proteins and lipids) and phenolic constituents from grains, which could stabilize or solubilize bioactive compounds and promote their accessibility to enzyme binding sites (Kieserling et al., 2024; Nocente & Gazza, 2025).

**Fig. 4:**
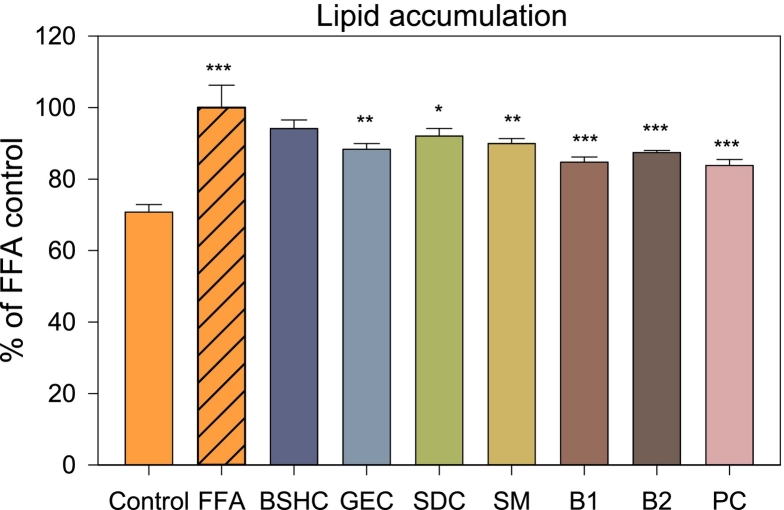
Inhibition of lipid accumulation in HepG2 cells under 500 μM free fatty acid (FFA)-induced steatosis conditions. The control group was untreated (normal control), whereas the FFA group was treated with FFA alone to induce lipid accumulation. Additional groups were treated with FFA combined with extracts (100 μg extract/mL) to assess lipid-lowering effects. BSHC, GEC, SDC, SM, B1, and B2 refer to Boseokheukchal, Goeunchal, Samdachal, Soman, optimized blend 1, and optimized blend 2, respectively. The positive control (PC) group was treated with coenzyme Q10. Values are expressed as percentages relative to the FFA-treated group (set at 100%) and are presented as mean ± SD (n = 3). *, **, and *** indicate statistically significant differences among treatments at *p* < 0.05, *p* < 0.01, and *p* < 0.001, respectively, according to one-way ANOVA followed by Tukey's HSD test.

The inhibitory effects of the optimized blends and their individual components on lipid accumulation were evaluated in the FFA-treated HepG2 cells (Fig. 4). FFA treatment markedly increased the intracellular lipid content compared to that in the untreated control (*p* < 0.001). Lipid accumulation was expressed as a percentage relative to the untreated control (set at 100%), where lower values indicate greater inhibitory effects. All extract-treated groups, except for BSHC, showed significantly reduced lipid accumulation relative to the FFA-treated group (*p* < 0.05), indicating their potential to attenuate hepatic lipid deposition. Among the individual components, GEC and SM exhibited the lowest lipid accumulation levels (88.32% and 89.94%, respectively), followed by SDC (92.02%) and BSHC (94.10%). In comparison, the PC treated with coenzyme Q10 showed a lipid accumulation level of 83.82%, serving as a benchmark for evaluating the effectiveness of the extracts. Notably, the optimized blends (B1 and B2) showed lower lipid accumulation levels (84.74% and 87.40%, respectively) than the individual components, indicating enhanced inhibitory effects. These results suggest that blending may enhance lipid metabolism-related bioactivity, which is consistent with the improved lipase inhibitory activity observed in vitro.

The enhanced lipid-lowering potential of the blended formulations may be associated with the complementary metabolic and phytochemical characteristics of the constituent grains and legumes (Huang et al., 2020; Mahboob et al., 2023). From the perspective of primary metabolites, SM exhibited superior carbohydrate and lipid indices, characterized by high dietary fiber and unsaturated fatty acid contents, which are favorable for lipid metabolism and oxidation. In contrast, SDC showed advantageous protein-related attributes, including a balanced EAA/NEAA ratio and abundant BCAA and SAA fractions, which are essential for hepatic lipid turnover and antioxidant defense. Regarding secondary metabolites, the blends benefited from the high extraction yield of SM and the rich phenolic and flavonoid profiles of GEC and BSHC, which contributed to their potent antioxidant and anti-inflammatory properties. Therefore, blending these nutritionally and functionally distinct materials yields a complementary combination of lipid-regulating, antioxidant, and enzyme-inhibitory properties, highlighting their potential as a multifunctional dietary formulation related to lipid metabolism. The enhanced bioactivity of the blends may involve, at least in part, dual mechanisms: (1) flavonoid-rich fractions inhibit pancreatic lipase and scavenge ROS, reducing substrate availability for hepatic triglyceride formation; and (2) primary metabolites, such as dietary fiber and free sugars, modulate FFA uptake and intracellular lipid handling. Taken together, these complementary pathways suppress hepatocellular lipid accumulation more effectively than the individual components.

### 3.6 Multivariate and correlation analysis

**Fig. 5:**
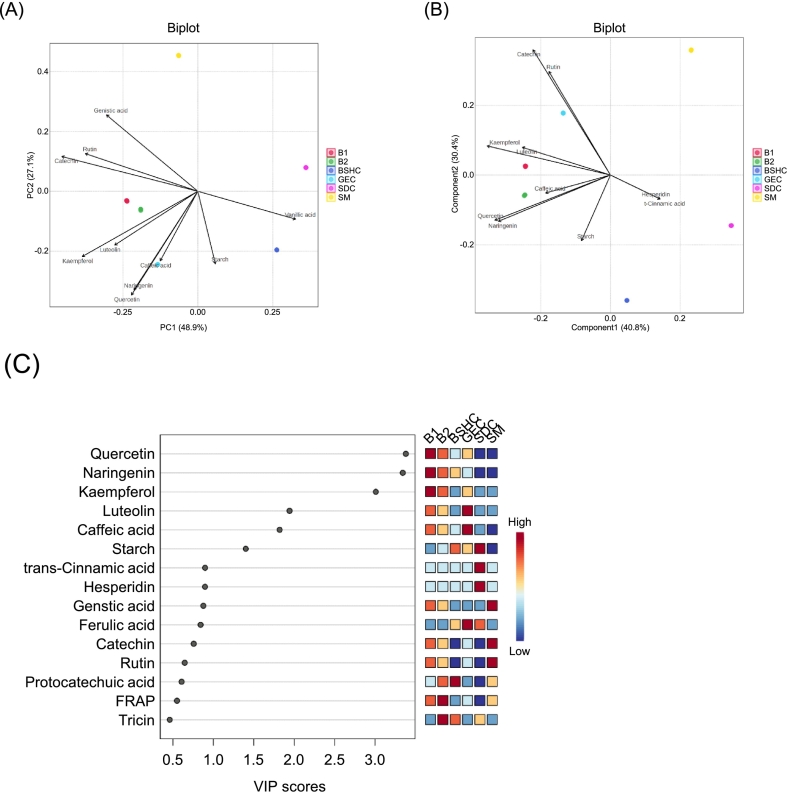

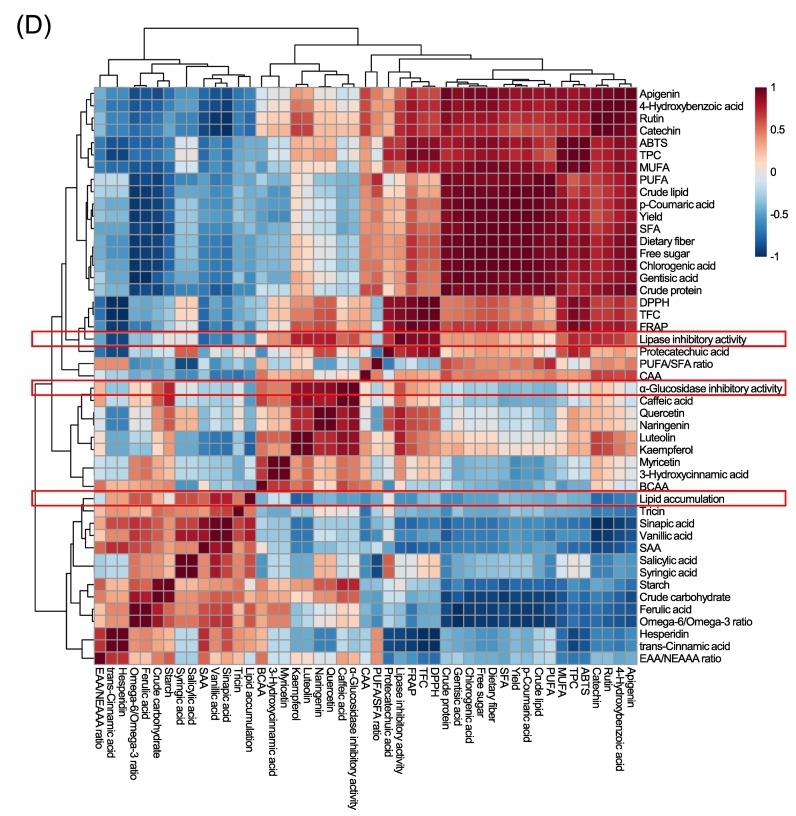

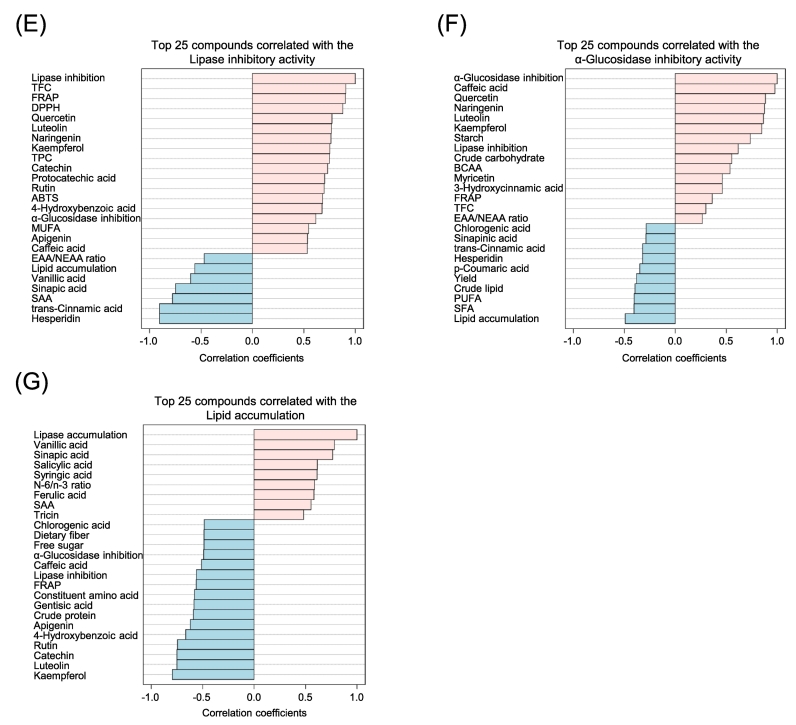
Multivariate and correlation analysis of metabolites and bioactivities. BSHC, GEC, SDC, SM, B1, and B2 refer to Boseokheukchal, Goeunchal, Samdachal, Soman, optimized blend 1, and optimized blend 2, respectively. (A) Principal component analysis (PCA) biplot showing the distribution of individual steamed grains and optimized blends based on primary and secondary metabolites. The top 10 contributing variables are displayed. (B) Partial least squares discriminant analysis (PLS-DA) biplot showing the separation among samples and the major contributing variables. The top 10 variables with the highest loadings are shown. (C) Variable importance in projection (VIP) scores from PLS-DA, indicating key metabolites responsible for group discrimination. (D) Pearson's correlation heatmap illustrating relationships among primary metabolites, phenolic compounds, and bioactivities. Red and blue colors represent positive and negative correlations, respectively. Red boxes highlight correlations associated with the inhibitory effects of lipase, α-glucosidase, and lipid accumulation. (E–G) PatternHunter analysis identifying the top 25 compounds most strongly correlated with the inhibitory effects of lipase (E), α-glucosidase (F), and lipid accumulation (G). (For interpretation of the references to color in this figure legend, the reader is referred to the web version of this article.)

Comprehensive multivariate analyses, including principal component analysis (PCA), partial least squares–discriminant analysis (PLS–DA), and correlation mapping, were conducted to elucidate the relationships between the compositional variables and biological activities using an integrated dataset of primary and secondary metabolites along with biological indices (Fig. 5). The PCA biplot (Fig. 5A) clearly distinguished the individual components and their optimized blends based on their metabolic profiles. Based on the loading plot (which illustrated only the top 10 variables in the biplot), PC 1 was primarily defined by high negative loadings of flavonoid-type compounds such as catechin, kaempferol, rutin, luteolin, quercetin, and naringenin, while hydroxybenzoic and hydroxycinnamic acids (vanillic acid, sinapic acid, and tiricin) contributed positively. PC 2 was mainly influenced by quercetin, caffeic acid, and naringenin on the negative side, and genistic acid, rutin, and catechin on the positive side. These distributions indicated that the separation of GEC and BSHC was driven by high levels of flavonoids and hydroxycinnamic acids, whereas SM was differentiated by benzoic acid derivatives and catechin-type compounds. SDC formed an independent cluster characterized by sinapinic acid and tricin. The optimized blends (B1 and B2) were positioned between these groups, reflecting the compositional convergence of legume- and cereal grain-derived phenolics through blending.

Building upon the PCA results, PLS–DA was subsequently applied to achieve clearer class separation and identify the variables most responsible for discrimination among the sample groups (Fig. 5B). The three-component PLS–DA model exhibited strong goodness-of-fit (R^2^ = 0.9597) and predictive capability (Q^2^ = 0.9371), indicating high model reliability. Cross-validation showed an increasing trend in both R^2^ and Q^2^ values as the number of components increased from one to three, suggesting that the model adequately captured the variance associated with the phenolic composition and bioactivity profiles among the samples. Distinct clustering of the individual components and optimized blends confirmed that the compositional characteristics were sufficient to discriminate between the groups. All variables were appropriately scaled prior to analysis, and model performance was evaluated using cross-validation to minimize the risk of overfitting. Therefore, the results should be interpreted as associative rather than causal. The variables important in projection (VIP) analysis (Fig. 5C) revealed that quercetin, naringenin, kaempferol, luteolin, caffeic acid, and starch were the major contributors (VIP > 1.0) to the discrimination pattern, consistent with the PCA results. These compounds were also strongly associated with antioxidant and lipase inhibitory activities, suggesting they may contribute to the observed bioactivity patterns.

Correlation and clustering analyses (Fig. 5D) revealed clear associations between phenolic profiles and biological activities. Flavonoid-type compounds were strongly correlated with antioxidant indices (DPPH, ABTS, and FRAP) and lipase inhibitory activity, indicating their key contribution to bioactive potential. In contrast, lipid accumulation formed an opposing cluster showing a negative correlation. These relationships suggest an inverse trend in which higher antioxidant and enzyme-inhibitory capacities are linked to lower lipid accumulation, reflecting a shared biochemical mechanism between oxidative defense and lipid metabolism regulation. Hierarchical clustering further demonstrated that the antioxidant- and enzyme-related parameters were grouped together, whereas lipid accumulation was positioned on the opposite axis, forming a contrasting bioactivity pattern. Collectively, these correlation structures support the idea that the enhanced bioactivity observed in the optimized blends may be associated with complementary interactions among compositional components. Correlation pattern analysis revealed a distinct association between the metabolic compounds and biological activity. Lipase inhibitory activity was strongly correlated with phenolic compounds, particularly flavonoids (Fig. 5E), whereas α-glucosidase inhibition was positively correlated with flavonoids, starch, crude carbohydrates, and BCAA (Fig. 5F). In contrast, lipid accumulation inhibition was more closely associated with crude protein, free sugars, and dietary fiber, indicating the contribution of primary metabolites to intracellular lipid regulation (Fig. 5G). These results suggest that both the phytochemical and nutritional components cooperatively contribute to the overall lipid-lowering efficacy of the blended formulations. Correlation patterns indicated that flavonoids primarily governed antioxidant and lipase inhibitory activities, whereas primary metabolites more strongly influenced intracellular lipid metabolism. This division of functional roles suggests that the lipid-lowering effect may arise from complementary interactions across metabolic pathways rather than from a single dominant constituent class. Such integrative behavior is characteristic of complex grains–legume matrices subjected to moist-heat processing.

## 4 Conclusion

This study demonstrates that integrating standardized steaming pretreatment with mixture design provides an effective strategy for optimizing compositional interaction and bioactivity in multigrain-legume systems. By minimizing variability among raw materials and applying a four-component mixture design, two optimized blends with high desirability were identified and experimentally validated, confirming the robustness of the predictive models. Black soybean and sorghum consistently contributed to enhanced extractability and bioactivity, while the inclusion of Italian millet or colored rice further differentiated the nutritional and phenolic characteristics of the blends. Despite these compositional differences, both optimized formulations exhibited comparable antioxidant and enzyme inhibitory activities, along with inhibitory effects on lipid accumulation. These findings highlight the importance of compositional complementarity in sharping functional outcomes and suggest that the proposed processing-informed mixture design framework may be useful for developing multifunctional grain-based ingredients with potential relevance to lipid metabolism.

## CRediT authorship contribution statement

**Narae Han:** Writing – original draft, Visualization, Validation, Software, Project administration, Methodology, Investigation, Formal analysis, Data curation, Conceptualization. **Moon Seok Kang:** Software, Methodology. **Hana Lee:** Writing – review & editing, Investigation, Data curation. **Jin Young Lee:** Investigation. **Yu-Young Lee:** Resources. **Junsoo Lee:** Writing – review & editing, Data curation. **Hyun-Joo Kim:** Writing – review & editing, Supervision, Resources, Project administration.

## Funding sources

This work was supported by the Cooperative Research Program for Agriculture Science and Technology Development (PJ017413022025) of the 10.13039/501100003627Rural Development Administration, Republic of Korea and the 2025 RDA Fellowship Program of the Department of Food Science, National Institute of Crop Science, Rural Development Administration, Republic of Korea.

## Declaration of competing interest

The authors declare that they have no known competing financial interests or personal relationships that could have appeared to influence the work reported in this paper.

## Data Availability

Data will be made available on request.

## Glossary

ABTS: 2,2′-azinobis(3-ethylbenzothiazoline-6-sulfonic acid)
ANOVA: analysis of variance
BCAA: branched-chain amino acid
BHT: butylated hydroxytoluene
CE: catechin equivalents
DMSO: dimethyl sulfoxide
DPPH: 2,2-diphenyl-1-picrylhydrazyl
DW: dry weight
EAA: essential amino acid
FAME: fatty acid methyl ester
FFA: free fatty acid
FRAP: ferric reducing antioxidant power
GAE: gallic acid equivalents
GC: gas chromatograph
HPLC: high-performance lipid chromatography
HSD: honest significance test
MUFA: monounsaturated fatty acid
NAFLD: nonalcoholic fatty liver disease
NEAA: non-essential amino acid
PC: positive control
PCA: principle component analysis
PLS-DA: partial least square discriminant analysis
PUFA: polyunsaturated fatty acid
ROS: reactive oxygen species
RSM: response surface methodology
SAA: sulfur-containing amino acid
SFA: saturated fatty acid
TE: Trolox equivalents
TFC: total flavonoid contents
TPC: total polyphenol contents
VIP: variables important in projection
