# Supplementary material for: Mixture design-based optimization of bioactivities in steamed grain and legume blends
Source: Food Chem X. 2026 Apr 22;36:103898. doi: 10.1016/j.fochx.2026.103898 (PMC13138058; doi:10.1016/j.fochx.2026.103898)
Supplement: Supplementary file 1 — Supplementary material. [file mmc1.docx]

**Supplementary Figure 1.** Changes in lipase inhibitory activity of steamed grains and legume samples at different steaming durations (0-50 min). BSHC, GEC, SDC, and SM refer to Boseokheukchal, Goeunchal, Samdachal, and Soman, respectively. Data are expressed as mean ± SD (n = 3). Different letter above bars within each cultivar indicate significant difference at *p* < 0.05 (Tukey’s HSD test).

**Supplementary Table 1.** ANOVA results for fitted models of extraction yield and enzyme inhibitory activities.

| Source | Yield (%) | | | | |  | Lipase (%) | | | | |  | α-Glucosidase (%) | | | | |
| --- | --- | --- | --- | --- | --- | --- | --- | --- | --- | --- | --- | --- | --- | --- | --- | --- | --- |
|  | Sum of  squares | df | Mean  squares | F-value | p-value |  | Sum of  squares | df | Mean  squares | F-value | p-value |  | Sum of  squares | df | Mean  squares | F-value | p-value |
| Block | 28.01 | 1 | 28.01 |  |  |  | 0.0004 | 1 | 0.0004 |  |  |  | 197.14 | 1 | 197.14 |  |  |
| **Model** | 182.79 | 3 | 60.93 | 6252.21 | < 0.0001 |  | 0.0016 | 9 | 0.0002 | 32.50 | < 0.0001 |  | 652.48 | 13 | 50.19 | 6.17 | 0.0174 |
| Linear Mixture | 182.79 | 3 | 60.93 | 6252.21 | < 0.0001 |  | 0.0012 | 3 | 0.0004 | 70.47 | < 0.0001 |  | 431.69 | 3 | 143.90 | 17.68 | 0.0022 |
| AB |  |  |  |  |  |  | 0.0000 | 1 | 0.0000 | 5.04 | 0.0486 |  | 31.45 | 1 | 31.45 | 3.86 | 0.0969 |
| AC |  |  |  |  |  |  | 2.086^E-06^ | 1 | 2.086^E-06^ | 0.3828 | 0.5499 |  | 25.29 | 1 | 25.29 | 3.11 | 0.1284 |
| AD |  |  |  |  |  |  | 0.0000 | 1 | 0.0000 | 6.32 | 0.0307 |  | 0.5629 | 1 | 0.5629 | 0.0692 | 0.8014 |
| BC |  |  |  |  |  |  | 0.0000 | 1 | 0.0000 | 3.45 | 0.0928 |  | 63.52 | 1 | 63.52 | 7.81 | 0.0314 |
| BD |  |  |  |  |  |  | 0.0003 | 1 | 0.0003 | 53.48 | < 0.0001 |  | 39.62 | 1 | 39.62 | 4.87 | 0.0695 |
| CD |  |  |  |  |  |  | 0.0001 | 1 | 0.0001 | 19.14 | 0.0014 |  | 5.58 | 1 | 5.58 | 0.6856 | 0.4394 |
| ABC |  |  |  |  |  |  |  |  |  |  |  |  | 10.27 | 1 | 10.27 | 1.26 | 0.3043 |
| ABD |  |  |  |  |  |  |  |  |  |  |  |  | 20.44 | 1 | 20.44 | 2.51 | 0.1641 |
| ACD |  |  |  |  |  |  |  |  |  |  |  |  | 4.72 | 1 | 4.72 | 0.5797 | 0.4753 |
| BCD |  |  |  |  |  |  |  |  |  |  |  |  | 38.81 | 1 | 38.81 | 4.77 | 0.0717 |
| **Residual** | 0.1559 | 16 | 0.0097 |  |  |  | 0.0001 | 10 | 5.450^E-06^ |  |  |  | 48.83 | 6 | 8.14 |  |  |
| Lack of fit | 0.1207 | 11 | 0.0110 | 1.56 | 0.3265 |  | 0.0000 | 5 | 4.559^E-06^ | 0.7189 | 0.6369 |  | 4.42 | 1 | 4.42 | 0.4971 | 0.5123 |
| Pure Error | 0.0352 | 5 | 0.0070 |  |  |  | 0.0000 | 5 | 6.341^E-06^ |  |  |  | 44.42 | 5 | 8.88 |  |  |
| **Cor Total** | 210.96 | 20 |  |  |  |  | 0.0021 | 20 |  |  |  |  | 898.46 | 20 |  |  |  |
| **Fit Statistics** |  |  |  |  |  |  |  |  |  |  |  |  |  |  |  |  |  |
| Std Dev^1)^ | 0.0987 |  |  |  |  |  | 0.0023 |  |  |  |  |  | 2.85 |  |  |  |  |
| Mean | 4.43 |  |  |  |  |  | 0.0354 |  |  |  |  |  | 16.09 |  |  |  |  |
| CV% | 2.23 |  |  |  |  |  | 6.59 |  |  |  |  |  | 17.73 |  |  |  |  |
| R^2^ | 0.9991 |  |  |  |  |  | 0.9669 |  |  |  |  |  | 0.9304 |  |  |  |  |
| Adjusted R^2^ | 0.9990 |  |  |  |  |  | 0.9372 |  |  |  |  |  | 0.7795 |  |  |  |  |
| Predicted R^2^ | 0.9985 |  |  |  |  |  | 0.8762 |  |  |  |  |  | −6.5447 |  |  |  |  |
| Adeq Precision^2)^ | 213.3609 |  |  |  |  |  | 20.6930 |  |  |  |  |  | 9.6640 |  |  |  |  |

^1)^Std Dev, standard deviation.

^2)^Adequate Precision (Adeq Precision) measures the signal to noise ratio.

**Supplementary Table 2.** Candidate formulation from the compositional optimization of grains-legume mixtures generated by Design-Expert software, with predicted responses and desirability scores.

| Number | Component 1:  BSHC^1)^  (%) | Component 2:  GEC  (%) | Component 3:  SDC  (%) | Component 4:  SM  (%) | Response 1:  Yield  (%) | Response 2:  Lipase  (%) | Response 3:  α-Glucosidase  (%) | Desirability |
| --- | --- | --- | --- | --- | --- | --- | --- | --- |
| **1^*^** | **0** | **38.835** | **15.978** | **45.187** | **6.250^2)^** | **34.065** | **33.340** | **0.806** |
| 2 | 0 | 40.957 | 15.668 | 43.375 | 6.064 | 34.418 | 33.341 | 0.806 |
| **3^*^** | **30.712** | **33.563** | **0** | **35.725** | **5.420** | **35.497** | **24.903** | **0.761** |
| 4 | 33.650 | 32.699 | 0 | 33.650 | 5.230 | 35.722 | 25.315 | 0.760 |
| 5 | 78.378 | 0 | 0 | 21.622 | 4.325 | 34.681 | 13.048 | 0.626 |
| 6 | 100 | 0 | 0 | 0 | 2.281 | 33.693 | 19.618 | 0.558 |

^*^The formulation was selected as the optimal blend.

^1)^BSHC, GEC, SDC and SM refer to Boseokheukchal, Goeunchal, Samdachal, and Soman, respectively.

^2)^All response values represented mean of replicates (n = 3).

**Supplementary Table 3.** Quantitative analysis of primary metabolites in individual components and optimized blends.

| Primary metabolites | BSHC^1)^ | GEC | SDC | SM | B1 | B2 |
| --- | --- | --- | --- | --- | --- | --- |
| *Proximate compositions (g/100 g DW)* | | | | | | |
| Ash | 1.45±0.05^2)^ | 1.39±0.02 | 1.64±0.05 | 5.52±0.01 | 3.20±0.06 | 2.81±0.04 |
| Protein | 7.77±0.18 | 13.21±0.16 | 10.11±0.24 | 38.33±0.20 | 24.96±0.43 | 20.92±0.45 |
| Carbohydrate | 88.00±0.13 | 82.98±0.17 | 84.37±0.17 | 35.55±0.29 | 60.75±0.42 | 67.03±0.51 |
| Lipid | 2.79±0.12 | 2.42±0.03 | 3.89±0.05 | 20.60±0.05 | 11.08±0.10 | 9.23±0.11 |
| *Constituent amino acid compositions (g/100 g protein)* | | | | | | |
| Histidine | 1.90±0.20 | 1.72±0.10 | 1.61±0.02 | 2.25±0.09 | 2.15±0.10 | 2.03±0.11 |
| Lysine | 3.24±0.32 | 1.26±0.08 | 1.62±0.11 | 5.59±0.21 | 4.43±0.23 | 4.13±0.26 |
| Phenylalanine | 4.43±0.31 | 4.33±0.32 | 4.41±0.16 | 4.24±0.15 | 4.52±0.21 | 4.04±0.23 |
| Threonine | 2.98±0.27 | 2.71±0.19 | 3.27±0.12 | 3.60±0.13 | 3.49±0.16 | 3.18±0.18 |
| Methionine | 1.04±0.10 | 0.99±0.12 | 1.91±0.10 | 0.91±0.05 | 1.01±0.08 | 0.92±0.07 |
| Isoleucine | 2.03±0.18 | 2.58±0.09 | 2.35±0.09 | 2.71±0.10 | 3.28±0.18 | 2.67±0.22 |
| Leucine | 6.04±0.50 | 11.92±1.09 | 9.82±0.41 | 6.25±0.22 | 8.20±0.40 | 7.21±0.39 |
| Valine | 3.42±0.28 | 3.55±0.11 | 3.26±0.08 | 2.94±0.11 | 3.73±0.19 | 3.10±0.21 |
| Alanine | 11.49±0.96 | 18.05±1.30 | 15.43±0.43 | 11.89±0.41 | 15.21±0.77 | 12.98±0.67 |
| Arginine | 25.08±2.02 | 29.06±2.10 | 28.25±0.72 | 28.48±1.04 | 30.79±1.54 | 27.27±1.42 |
| Aspartic acid | 4.52±0.42 | 8.56±0.65 | 7.00±0.40 | 3.83±0.14 | 4.98±0.26 | 4.66±0.31 |
| Glutamic acid | 5.27±0.52 | 2.06±0.12 | 2.30±0.07 | 6.01±0.24 | 4.88±0.25 | 4.75±0.31 |
| Cysteine | 7.68±0.72 | 5.32±0.42 | 5.65±0.31 | 10.60±0.39 | 9.03±0.42 | 8.61±0.59 |
| Glycine | 12.84±1.15 | 18.93±1.42 | 16.17±0.76 | 16.19±0.56 | 16.90±0.80 | 15.46±0.96 |
| Proline | 2.27±0.08 | 1.52±0.12 | 1.81±0.05 | 1.58±0.05 | 1.73±0.04 | 1.32±0.07 |
| Serine | 3.90±0.36 | 2.25±0.12 | 2.33±0.13 | 3.86±0.14 | 3.36±0.15 | 3.35±0.22 |
| Tyrosine | 3.05±0.45 | 7.77±0.36 | 5.51±0.37 | 4.31±0.17 | 4.84±0.29 | 4.59±0.31 |
| *Carbohydrate compositions (g/100 g DW)* | | | | | | |
| Starch | 83.13±1.61 | 71.84±2.13 | 83.37±1.74 | 0.13±0.01 | 42.67±2.01 | 49.74±1.81 |
| Soluble fiber | 6.40±0.38 | 6.45±0.09 | 4.86±0.06 | 27.72±1.04 | 18.68±0.60 | 15.30±0.22 |
| Insoluble fiber | 0.36±0.02 | 0.11±0.02 | 0.16±0.04 | 4.26±0.57 | 0.66±0.05 | 0.60±0.07 |
| Fructose | nd | nd | nd | 0.30±0.01 | 0.15±0.01 | 0.11±0.01 |
| Glucose | 0.16±0.00 | 0.14±0.01 | 0.19±0.00 | 0.15±0.01 | 0.17±0.02 | 0.15±0.00 |
| Sucrose | 2.70±0.01 | 1.00±0.02 | 1.35±0.03 | 0.65±0.03 | 3.20±0.04 | 3.19±0.06 |
| Raffinose | 0.02±0.00 | 0.10±0.01 | 0.15±0.01 | 0.42±0.01 | 0.22±0.02 | 0.15±0.00 |
| Stachyose | nd | nd | nd | 5.67±0.02 | 2.91±0.03 | 2.36±0.05 |

(Continued)

| *Fatty acid composition (mg FAME/100 g DW)* | | | | | | |
| --- | --- | --- | --- | --- | --- | --- |
| Capric acid (C10:0) | 3.66±0.28 | 3.26±0.05 | 3.64±0.14 | nd | nd | nd |
| Lauric acid (C12:0) | 2.23±0.17 | 1.93±0.11 | nd | nd | nd | nd |
| Myristic acid (C14:0) | 20.14±1.28 | 2.79±0.21 | 3.11±0.15 | 20.47±1.15 | 9.62±0.30 | 12.63±0.35 |
| Pentadecanoic acid (C15:0) | 2.13±0.10 | 1.33±0.11 | 2.60±0.20 | 4.30±0.30 | 2.62±0.08 | 2.31±0.03 |
| Palmitic acid (C16:0) | 474.29±33.14 | 456.11±30.54 | 261.87±23.50 | 1821.96±130.91 | 879.91±48.42 | 826.54±26.71 |
| Heptadecanoic acid (C17:0) | 2.40±0.14 | 2.94±0.22 | 3.66±0.31 | 16.25±1.04 | 8.06±0.28 | 6.99±0.38 |
| Stearic acid (C18:0) | 57.94±9.06 | 51.73±4.41 | 142.04±13.62 | 577.56±43.22 | 265.56±14.93 | 217.55±5.95 |
| Arachidic acid (C20:0) | 18.29±1.40 | 6.96±0.38 | 40.90±4.36 | 62.70±4.53 | 34.00±1.84 | 27.26±0.86 |
| Heneicosanoic acid (C21:0) | nd | nd | 3.29±0.24 | 9.62±0.49 | 4.90±0.13 | 3.95±0.14 |
| Behenic acid (C22:0) | 7.97±0.62 | 4.51±0.33 | 18.17±1.72 | 81.14±6.86 | 36.81±2.30 | 30.58±1.08 |
| Tricosanoic acid (C23:0) | nd | nd | 4.01±0.26 | 12.68±1.03 | 6.58±0.11 | 5.71±0.48 |
| Lignoceric acid (C24:0) | 15.14±1.26 | 6.21±0.47 | 8.89±0.72 | 33.94±2.49 | 17.30±0.71 | 18.18±0.38 |
| Palmitoleic acid (C16:1 cis-9) | 6.11±1.03 | 8.80±0.42 | 2.71±0.14 | 19.33±1.30 | 10.73±0.48 | 10.49±0.24 |
| cis-10-Heptadecenoic acid (C17:1) | 1.27±0.11 | nd | nd | 11.76±0.61 | 5.00±0.31 | 4.55±0.23 |
| Eladic acid (C18:1 trans-9) | nd | nd | 1.69±0.11 | 8.64±0.62 | 4.39±0.38 | 3.97±0.06 |
| Oleic acid (C18:1 cis-9) | 1070.35±80.58 | 811.57±20.48 | 355.15±36.18 | 4144.61±306.70 | 1918.36±110.78 | 1834.73±59.71 |
| cis-11-Eicosenoic acid (C20:1) | 13.90±1.04 | 5.82±0.13 | 9.75±0.93 | 37.48±2.71 | 18.30±0.87 | 17.74±0.07 |
| Erucic acid (C22:1 cis-13) | 1.32±0.07 | nd | nd | nd | nd | nd |
| Linoleic acid (C18:2 cis-9,12) | 900.41±64.10 | 1280.53±36.60 | 1886.73±188.57 | 8733.37±622.98 | 4113.87±228.20 | 3410.92±108.87 |
| γ-Linolenic acid (C18:3 cis-6,9,12) | nd | nd | 1.51±0.07 | 6.64±0.27 | 3.31±0.07 | 2.88±0.03 |
| α-Linolenic acid (C18:3 cis-9,12,15) | 36.96±2.60 | 54.76±2.72 | 85.87±8.21 | 1043.23±72.72 | 441.34±24.05 | 364.98±11.79 |
| cis-11,14-Eicosadienoic acid (C20:2) | 1.54±0.06 | 0.99±0.07 | 2.73±0.22 | 12.77±1.04 | 5.81±0.29 | 4.76±0.12 |

^1)^BSHC, GEC, SDC, SM, B1, and B2 refer to Boseokheukchal, Goeunchal, Samdachal, Soman, optimized blend 1, and optimized blend 2, respectively.

^2)^All response values represented mean of replicates (n = 3). nd, not detected.

**Supplementary Table 4.** Quantitative analysis of individual phenolic compounds (mg/100 g dry weight) identified in individual components and optimized blends.

| Phenolic compounds | BSHC^1)^ | GEC | SDC | SM | B1 | B2 |
| --- | --- | --- | --- | --- | --- | --- |
| *Hydroxy benzoic acids* |  |  |  |  |  |  |
| Protocatechuic acid | 57.64 ± 0.59^2)^ | 7.97 ± 0.14 | 0.53 ± 0.01 | 12.33 ± 0.27 | 9.09 ± 0.01 | 25.08 ± 0.21 |
| 4-Hydroxybenzoic acid | 1.03 ± 0.04 | 3.19 ± 0.05 | 0.70 ± 0.06 | 8.80 ± 0.12 | 6.05 ± 0.17 | 4.60 ± 0.06 |
| Gentisic acid | nd^1)^ | nd | nd | 18.96 ± 0.22 | 8.61 ± 0.36 | 6.81 ± 0.19 |
| Vanillic acid | 12.25 ± 0.07 | nd | 0.91 ± 0.01 | nd | nd | nd |
| Syringic acid | 0.26 ± 0.01 | nd | nd | nd | nd | nd |
| Salicylic acid | 5.84 ± 0.01 | nd | nd | nd | nd | nd |
| Subtotal | 77.03 ± 0.69 | 11.16 ± 0.20 | 2.14 ± 0.06 | 40.09 ± 0.60 | 23.74 ± 0.44 | 36.49 ± 0.22 |
| *Hydroxycinnamic acids* |  |  |  |  |  |  |
| Chlorogenic acid | 7.48 ± 0.00 | 7.54 ± 0.07 | 6.66 ± 0.01 | 37.53 ± 0.06 | 22.57 ± 0.06 | 18.71 ± 0.03 |
| Caffeic acid | 0.18 ± 0.00 | 7.71 ± 0.12 | 0.17 ± 0.01 | nd | 3.44 ± 0.21 | 2.34 ± 0.27 |
| 3-Hydroxycinnamic acid | nd | 4.03 ± 0.00 | nd | nd | nd | nd |
| p-Coumaric acid | 6.71 ± 0.01 | 4.92 ± 0.14 | 5.52 ± 0.03 | 31.60 ± 0.03 | 18.25 ± 0.05 | 15.28 ± 0.01 |
| Sinapic acid | 0.26 ± 0.00 | nd | 0.24 ± 0.00 | nd | nd | nd |
| Ferulic acid | 1.00 ± 0.02 | 1.8 ± 0.01 | 1.20 ± 0.02 | nd | nd | nd |
| trans-Cinnamic acid | nd | nd | 2.11 ± 0.00 | nd | nd | nd |
| Subtotal | 15.68 ± 0.03 | 26.08 ± 0.30 | 15.90 ± 0.01 | 69.14 ± 0.07 | 44.26 ± 0.26 | 36.33 ± 0.26 |
| *Flavonoids* |  |  |  |  |  |  |
| Catechin | nd | 20.01 ± 0.18 | nd | 55.21 ± 1.10 | 34.92 ± 0.59 | 27.36 ± 0.20 |
| Hesperidin | nd | nd | 2.10 ± 0.00 | nd | nd | nd |
| Naringenin | 8.47 ± 0.03 | 5.91 ± 0.02 | nd | nd | 22.82 ± 0.01 | 19.20 ± 0.04 |
| Myricetin | nd | 7.43 ± 0.03 | nd | nd | nd | nd |
| Quercetin | 10.17 ± 0.03 | 10.88 ± 0.03 | nd | nd | 26.31 ± 0.02 | 22.40 ± 0.03 |
| Kaempferol | nd | 8.77 ± 0.03 | nd | nd | 26.23 ± 0.03 | 21.92 ± 0.08 |
| Rutin | nd | 2.23 ± 0.06 | nd | 16.70 ± 0.15 | 9.48 ± 0.13 | 7.50 ± 0.16 |
| Luteolin | nd | 3.99 ± 0.03 | nd | nd | 1.71 ± 0.04 | 1.39 ± 0.03 |
| Apigenin | 2.69 ± 0.02 | 5.53 ± 0.06 | 2.51 ± 0.01 | 14.89 ± 0.04 | 9.97 ± 0.01 | 7.10 ± 0.14 |
| Tricin | 3.34 ± 0.00 | nd | 2.40 ± 0.03 | nd | nd | 5.92 ± 0.10 |
| Subtotal | 24.67 ± 0.02 | 64.75 ± 0.31 | 7.01 ± 0.02 | 86.80 ± 1.22 | 131.44 ± 0.61 | 112.80 ± 0.30 |
| Total | 117.38 ± 0.69 | 101.99 ± 0.75 | 25.06 ± 0.07 | 196.02 ± 1.69 | 199.44 ± 1.18 | 185.62 ± 0.49 |

^1)^BSHC, GEC, SDC, SM, B1, and B2 refer to Boseokheukchal, Goeunchal, Samdachal, Soman, optimized blend 1, and optimized blend 2, respectively.

^2)^All response values represented mean of replicates (n = 3). nd, not detected.
